# Plasma cytokine levels and PCOS risk: Mendelian randomization analysis reveals IL6R as a preventive factor

**DOI:** 10.1186/s13048-025-01647-w

**Published:** 2025-04-03

**Authors:** Xiaopei Li, Yan Li, Bumei Zhang, Jianmei Wang, Yang Yang, Yongrui Du

**Affiliations:** 1 Department of Family Planning, The Second Hospital of Tianjin Medical University, Tianjin, China; 2 Department of Bioinformatics, School of Basic Medical Sciences, Tianjin Medical University, Tianjin, China; 3 Department of Pharmacology, School of Basic Medical Sciences, Tianjin Key Laboratory of Inflammation Biology, Tianjin Medical University, Tianjin, China

**Keywords:** Two-sample Mendelian randomization, Polycystic ovary syndrome, Cytokines, GWAS, IL6R

## Abstract

**Background:**

Polycystic ovary syndrome is a prevalent gynecological condition affecting primarily women of childbearing age. It is characterized by elevated androgen levels, ovulatory dysfunction, and morphological abnormalities. Despite extensive research from various perspectives, the etiology and pathogenesis of PCOS remain unclear. While controversial, many believe that individuals with PCOS exhibit a chronic low-grade inflammatory state. Cytokines play diverse roles in the initiation and progression of inflammation, contributing to this inflammatory milieu. Therefore, the aim of this study was to utilize publicly available genome-wide association study data to explore the potential causal relationship between cytokines and PCOS.

**Methods:**

To accurately investigate the causal relationship between cytokines and PCOS, we initially defined cytokines using the GeneCrad and then identified cytokines in two independent large-scale plasma proteins. Subsequently, we employed a two-sample Mendelian randomization analysis framework. A series of quality control procedures were implemented to select eligible instrumental variables closely associated with the exposure. MR analysis was conducted using genome-wide association studies of PCOS in two independent European ancestry groups. Cochran, s Q test, MR-Egger and intercept test were employed to assess heterogeneity and pleiotropy in PCOS. Co-localization analysis, summary-data-based Mendelian randomization analysis, and HEIDI testing were utilized to further corroborate the relationship between positive findings and PCOS. Finally, systematical Mendelian randomization analysis between healthy lifestyle factors and PCOS-related proteins was conducted to identify which proteins could act as interventional targets by lifestyle changes.

**Results:**

In our investigation, we performed Mendelian randomization analysis on 33 cytokines in relation to PCOS using data from the deCODE and the Fenland. Our findings revealed that the plasma level of IL6R emerges as a notable protective factor against PCOS, exhibiting a substantial effect size. Moreover, we identified CCL22 as a significant risk factor for PCOS, a finding that was similarly validated and supported by independent cohorts.

**Conclusion:**

Our Mendelian randomization analysis, leveraging genome-wide association study data from a sizable population cohort, unequivocally delineated a causal relationship between IL6R and PCOS. These results underscore the involvement of cytokines in the pathogenesis of PCOS and highlight their potential as promising therapeutic targets for addressing this intricate disease.

**Supplementary Information:**

The online version contains supplementary material available at 10.1186/s13048-025-01647-w.

## Background

Polycystic ovary syndrome (PCOS) is characterized by elevated androgen levels, ovulatory dysfunction, and morphological abnormalities, predominantly affecting women aged 15 to 49 years [1, 2]. Its incidence among women of childbearing age ranges from 6 to 10% [3]. Diagnosis according to the 2003 ESHRE/ASRM criteria requires the presence of any two out of three criteria: (1) oligo/anovulation, (2) hyperandrogenemia, and (3) polycystic ovaries. Women with PCOS often experience serious complications such as infertility, irregular menstrual cycles, as well as metabolic dysfunctions like obesity and long-term cardiovascular disease [4–6].

Various research efforts have been undertaken from different perspectives to better understand PCOS for improved prevention and management strategies. A review published in Nature Reviews Endocrinology provides key insights into the etiology and pathophysiology of PCOS [7–11]. In ancient times when diets lacked sufficient proteins and fats, the combination of thrifty genotypes and phenotypes may have been essential for survival. However, with an adequate food supply the defensive mechanisms of thrifty genotypes are no longer beneficial in recent decades, leading to a significant increase in the prevalence of obesity and diabetes [12–14]. Similarly, a theory suggesting that insulin resistance may play a major role in thrifty genotypes and phenotype has been proposed for PCOS [12]. A view also highlights familial aggregation, androgen excess, and insulin resistance in relation to PCOS [13, 15]. In conclusion, PCOS is now considered a complex multi-genetic disorder in terms of etiology. Predisposing and protective genetic variants interact with strong environmental influences resulting in different PCOS phenotypes, which is similar to other prevalent metabolic disorders such as type 2 diabetes mellitus [16].

Considering genetic factors and lifestyle choices contribute to the development of PCOS, numerous studies have highlighted the significant association between PCOS and immune responses. Immune cells and their secreted cytokines play a crucial role in the pathogenesis of PCOS. PCOS is often accompanied by a pro-inflammatory state, which may contribute to ovulation failure and subsequent infertility [10]. Several studies have indicated that PCOS is associated with elevated levels of cytokines such as IL-18, MCP-1 (monocyte chemoattractant protein-1), and others [17–23]. Particularly, IL-18, a pro-inflammatory cytokine, is closely linked to insulin resistance and metabolic syndrome [24]. Elevated IL-18 concentrations have been observed in PCOS patients, regardless of insulin resistance and obesity status [18]. Additionally, hyperglycemia in PCOS, resulting from obesity and insulin resistance, can further exacerbate the inflammatory processes. Disordered granulosa cells in the ovaries of PCOS patients may activate inflammation, potentially leading to mitochondrial damage. Consequently, the altered follicular microenvironment can affect granulosa cell function which results in impaired development and growth of oocytes [10].

Several studies have reported an association between PCOS and low-grade chronic inflammation [24]. They found that women with PCOS exhibited lower levels of C-reactive protein (CRP), interleukin 18 (IL-18), tumor necrosis factor (TNF-α), interleukin 6 (IL-6), and other markers such as white blood cell (WBC) count, MCP-1, and macrophage inflammatory protein-1α (MIP-1α). Additionally, numerous factors related to oxidative stress, inflammation, and thrombosis (e.g., asymmetric dimethylarginine (ADMA), homocysteine, plasminogen activator inhibitor-I (PAI-I), vascular endothelial growth factor (VEGF) have been proposed as cardiovascular risk markers indicating endothelial cell damage in PCOS. Recent research has also highlighted the significant relationship between PCOS and immune response [25–27]. Various cytokines have been implicated in the development of PCOS, but opinions on this matter vary.

Observational studies, however, may be susceptible to confounding factors and reverse causation, which diminishes their credibility. Residual confounding factors and potential reverse causation presenting in traditional observational studies pose significant challenges in measuring the causal impact of specific biomarkers on PCOS risk. Mendelian randomization (MR), using genetic variation as an instrumental variable for the exposure of interest, offers a novel statistical method for examining causal relationships between exposures and outcomes [28, 29]. By leveraging of randomly assigned genetic variants during gamete formation and conception, MR analysis can mitigate confounding bias and reverse causal relationships. While MR analysis has been widely employed to determine causality between factors and health outcomes, the relationship between cytokines in plasma proteins and PCOS has not been studied through Mendelian randomization (MR) analysis up to now [30, 31]. Hence, we conducted a large-scale MR study supplemented by colocalization, pooled data-based MR (SMR), and HEIDI testing to identify underlying mechanisms and novel causal cytokines in PCOS.

## Methods

### Study design

**Fig. 1:**
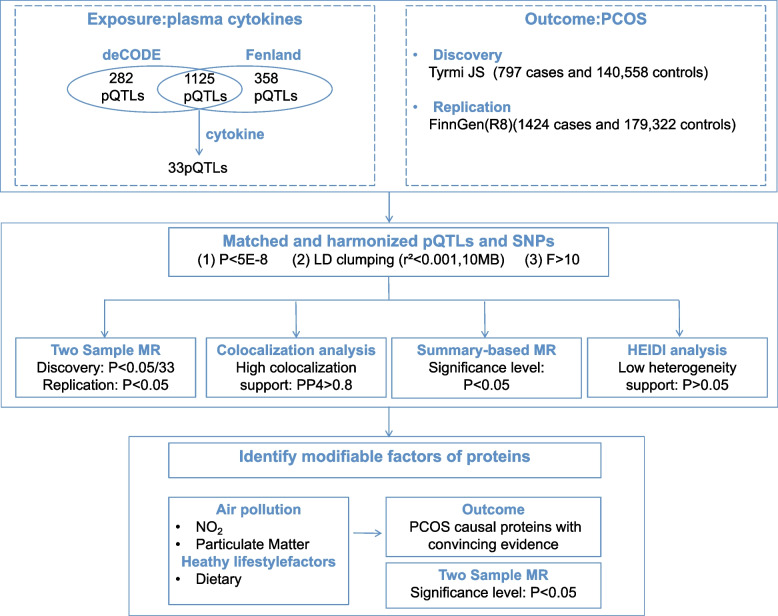
Study design. This flowchart illustrates the comprehensive research design, data sources, and methodologies utilized in the study

The flow diagram illustrating the methodology of the study is presented in Fig. 1. Initially, we intersected two large-scale plasma protein GWAS datasets, resulting in a shared set of 1,125 plasma proteins. Using the GeneCrad database, we applied the "cytokine" filter and identified cytokines with scores above 7. The threshold of 7 was determined by selecting the top 12.5% of the highest scoring cytokines in the GeneCrad database and rounding to the nearest integer. This cutoff was chosen to retain cytokines with strong biological evidence while filtering out less relevant associations. This set was then intersected with the plasma protein data to serve as exposure variables. Instrumental variables were extracted for each protein across different cohorts, and two-sample Mendelian Randomization analyses were conducted separately using PCOS data from the Tyrmi JS study and FinnGen. Subsequently, we categorized the results and performed co-localization analysis on validated proteins to pinpoint potential drug targets. Finally, we employed the SMR method to reaffirm the causal relationship between plasma proteins and PCOS.

Mendelian Randomization analysis relies on three crucial assumptions. Firstly, the genetic variation must be significantly associated with the exposure. Secondly, the genetic variation used as an instrumental variable for the exposure must be independent of other confounding factors. Lastly, the genetic variation should influence outcomes solely through its effect on the exposure.

### Genetic instrumental variables selection

Cytokines were derived from genome-wide association studies (GWAS) conducted on European populations, as reported by the deCODE study in Iceland and the Fenland study. Rigorous quality control measures were meticulously applied to each cytokines, and we carefully curated eligible instrumental variables (IVs). Specifically, candidate IVs for subsequent analysis were identified among plasma proteins with single nucleotide polymorphisms (SNPs) that reached genome-wide significance (*P* < 5e-8). To ensure the independence of each IV for the exposure, LD-based clumping was employed to eliminate SNPs in strong linkage disequilibrium (LD) (with an r2 threshold of 0.1 and a window size of 10,000 kb). Additionally, to mitigate weak instrumental variable bias, the F-statistic was computed to assess the strength of the IV. An F-statistic exceeding 10 indicated a reduced likelihood of weak instrumental variable bias, and IVs with F-statistics > 10 were selected for further Mendelian randomization analysis.

### GWAS summary statistics of Polycystic ovary syndrome

The genome-wide association study (GWAS) summary statistics were sourced from the study by Tyrmi JS et al., comprising 797 cases and 140,558 controls [32]. Additionally, another GWAS on PCOS was from FinnGen R8, which focused on individuals of European ancestry, were obtained from the official website (https://r8.finngen.fi/). These summary statistics for PCOS included data from 1424 cases and 179,322 controls.

### Mendelian randomization estimates

We integrated the summary statistics to independently estimate the causal relationships between each plasma protein and PCOS using various MR methods, including inverse variance weighting (IVW), weighted mean (WM), MR-Egger, and Wald ratio (for models with only 1 SNP). The IVW method served as the primary statistical model, with both fixed effects and random effects IVW methods available.

Initially, we computed the causal estimates using the fixed effects IVW method by meta-analyzing Wald ratio estimates for each instrumental variable. If significant heterogeneity (*P* < 0.05) was observed, we introduced the random effects IVW method. Adhering to the three critical assumptions fundamental to the MR approach is vital to ensure the reliability of the obtained causal estimates. Heterogeneity in causal estimates across IVs may imply non-compliance with these assumptions. We employed Cochran's Q test to detect heterogeneity, utilizing both the causal estimates from the fixed effects IVW method and the MR-Egger regression [33]. Cochran's Q statistics were employed to quantify heterogeneity, and we considered statistical significance at a *P*-value < 0.05.

To evaluate the possibility of pleiotropic effects of IVs, we utilized the MR-Egger regression approach, which can indicate the presence of directional horizontal pleiotropic effects in the causal estimates based on the intercept term. Additionally, we conducted a leave-one-out analysis, systematically excluding each SNP in turn, and subsequently ran the MR analysis on the remaining SNPs to identify any potentially outlying instrumental variables.All MR analyses were conducted using the R package TwoSampleMR (version 0.5.6) [34].

### Colocalization analysis

To evaluate whether there are common causal variants shared between the signals of IL6R and PCOS risk, we conducted a colocalization analysis using summary statistics from IL6R and PCOS genome-wide association studies (GWAS). This analysis, implemented through the "coloc" package, involved testing five hypotheses:

(i) No causal variant exists in either IL6R or PCOS loci (H0). (ii) A single causal variant exists in IL6R only (H1). (iii) A single causal variant exists in PCOS only (H2). (iv) IL6R and PCOS have distinct causal variants (H3). (v) IL6R and PCOS share a common causal variant (H4) [35].

For IL6R, we considered SNPs within ± 5000 kb of the pQTL. The colocalization analysis utilized default parameters with priors set as follows: p1 = 1 × 10 − 4 (prior probability of SNP association with the protein), p2 = 1 × 10 − 4 (prior probability of SNP association with PCOS), and p12 = 1 × 10 − 5 (prior probability of SNP association with both IL6R and PCOS) [35]. Given the sensitivity of colocalization to priors and window sizes, we performed additional analyses with altered priors (p12 = 1e − 6) to validate the robustness of our findings. Posterior probabilities were calculated to quantify support for each hypothesis, with posterior probabilities of H4 (PP4) exceeding 80% under different priors and windows considered indicative of strong evidence for colocalization.

### Summary-data-based MR (SMR) analysis

Summary-data-based MR (SMR) analysis was further conducted as a complementary method to verify the causal associations between IL6R and PCOS. The heterogeneity in dependent instruments (HEIDI) test, using multiple SNPs in a region, was employed to distinguish proteins that were associated with PCOS risk owing to a shared genetic variant rather than genetic linkage. The SMR and HEIDI tests were performed using SMR software (SMR v1.3.1). A *P* value < 0.05 was defined as the significance level for SMR [36]. A *P* value of > 0.05 for the HEIDI test indicated that the association of IL6R and PCOS was not driven by linkage disequilibrium.

### Healthy lifestyle factors of PCOS-related proteins

Additionally, we conducted Mendelian randomization analyses of healthy lifestyle factors with PCOS-related proteins to identify which PCOS-related proteins could be modulated by healthy lifestyle interventions. We considered all lifestyle and air pollution factors available in the UK Biobank. However, after applying instrumental variable selection criteria, including the presence of genome-wide significant SNPs and sufficient F-statistics to avoid weak instrument bias, only 14 healthy lifestyle factors and 3 air pollution factors remained eligible for MR analysis. A total of 14 healthy lifestyle and 3 air pollutions factors (Additional file 1: Table S11) were employed to evaluate their associations with convincing PCOS causal proteins. The analytical methods for the Mendelian randomization were consistent with the description provided in the two sample Mendelian randomization analysis.

## Results

### Selection of cytokines in plasma proteins

We initially intersected two large-scale plasma protein GWAS datasets from deCODE and Fenland, and obtained a shared set of 1,125 plasma proteins. In the GeneCrad database, we applied the "cytokine" qualification and screened for cytokines with relevant scores above 7. Consequently, we identified a subset of 33 cytokines with significant associations as plasma protein quantitative trait loci (pQTLs), which were subsequently utilized as exposure factors for further analysis.

### Selection of instrumental variables

We systematically selected 33 cytokine pQTLs from genome-wide association studies (GWAS) of plasma proteins. These cytokines were categorized into four groups: interleukin factors (IL6R, IL6ST, IL10RB, IL12B, IL1RN, IL16, IL2RB, IL18R1, IL22RA2, IL12RB1, IL1R1, IL17RA, IL27RA, EBI3), chemokines (CCL22, CCL5, CCL17, CCL11, CCL3, CCL18, CXCL5), tumor necrosis factor (TNFSF12, TNFRSF11B), and others (STAT3, STAT6, CYTL1, ADIPOQ, CD14, VEGFA, IFNAR1, TLR3, LIFR, CNTFR).

We initially retained single nucleotide polymorphisms (SNPs) significantly associated with each exposure phenotype in the respective GWAS study (*P* < 5e − 8). Subsequently, we performed LD-based clumping to obtain LD-independent SNPs for each exposure, employing an r^2^ threshold of 0.1 and a window size of 10 Mb. It was imperative to ensure that the effect of an SNP on both the exposure and the outcome was attributed to the same allele. During the harmonization process, ambiguous SNPs with non-concordant alleles and palindromic SNPs with ambiguous strands that could not be resolved were discarded. Consequently, the number of SNPs chosen as instrumental variables for each exposure in subsequent two-sample Mendelian randomization (MR) analyses equaled or was less than the count listed in Additional file 1: Table S1&S2. To evaluate the strength of each instrumental variable, we computed the F-statistics for each instrument-exposure association. Notably, the F-statistics obtained were substantially greater than 10, indicating that these SNPs served as robust instrumental variables (Additional file 1: Table S1 & S2).

### Causal effects of cytokines on PCOS (discovery)

**Fig. 2:**
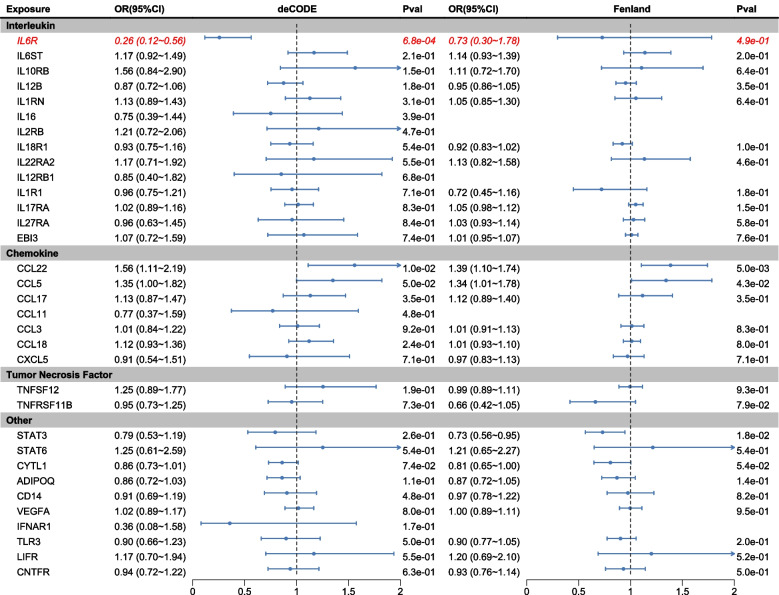
Summary of MR estimates of cytokine (deCODE) on the risk of PCOS. Forest plots illustrating the causal relationships and effect sizes between various types of cytokines from the deCODE database and PCOS. The OR (OR) was calculated using the fixed effect IVW method and Wald Ratio, with the horizontal bars indicating 95% confidence intervals (CI). OR values are represented by squares, and their corresponding confidence intervals are depicted by horizontal lines. If the confidence intervals fall outside the range of 0.5–1.5, they are indicated by arrows

We conducted a two-sample MR analysis to evaluate the effects of 33 cytokines, using data from the deCODE and Fenland database for cytokine pQTLs as exposure factors and the GWAS summary statistics of PCOS patients from European ancestry provided by Tyrmi JS et al. The MR estimates in different methods are detailed in Table S3 and S5 of the supplementary files 1. Our results revealed a significant causal association between genetically predicted plasma IL6R levels and reduced PCOS risk (IVW: OR = 0.26, 95% CI: 0.12–0.56, *P* = 6.38e − 04) (Fig. 2 and Additional file 1: Table S3). Notably, this significant negative causal effect of plasma IL6R levels on PCOS persisted even after adjusting for multiple comparisons of the 33 cytokines (IVW: adjusted *P* = 2.32e − 02) (Additional file 1: Table S3). Additionally, we identified a positive causal relationship between genetically predicted CCL22 levels and PCOS (IVW: OR = 1.56, 95% CI: 1.11–2.19, *P* = 1.01e − 2) (Fig. 2 and Additional file 1: Table S3). However, in the deCODE analysis, no other cytokines demonstrated a significant causal effect on PCOS (Fig. 2 and Additional file 1: Table S3). Heterogeneity testing indicated that the above two cytokines, IL6R and CCL22, were not heterogeneous in PCOS analysis (IL6R: IVW Cochran's Q = 0.608, P_heterogeneity_ = 0.435; CCL22: IVW Cochran's Q = 3.251, P_heterogeneity_ = 0.071) (Additional File 1: Table S4). To mitigate potential data biases, we undertook another analysis selecting 33 cytokines from Fenland's plasma protein cohort as exposure variables for MR analysis. Employing two-sample MR analysis, we juxtaposed cytokine pQTLs from the Fenland with PCOS GWAS summary statistics from Tyrmi JS et al. Regrettably, in this analysis, plasma IL6R levels remained a protective factor for PCOS but did not achieve statistical significance (WR: OR = 0.73, 95% CI: 0.30–1.78, *P* = 4.86e − 01) (Fig. 2 and Additional file 1: Table S5). Nevertheless, our analysis reaffirmed a significant positive causal association between plasma CCL22 levels and PCOS risk (IVW: OR = 1.39, 95% CI: 1.10–1.74, *P* = 4.99e − 03). Furthermore, plasma CCL5 levels exhibited a notable association with PCOS onset (WR: OR = 1.34, 95% CI: 1.01–1.78, *P* = 4.26e − 02) (Fig. 2 and Additional file 1: Table S5). Conversely, a reverse causal relationship was observed between genetically predicted plasma STAT3 levels and PCOS (WR: OR = 0.73, 95% CI: 0.56–0.95, *P* = 1.76e − 02) (Fig. 2 and Additional file 1: Table S5).

In summary, our findings indicate that the association between IL6R levels and reduced PCOS risk has been consistently verified across different exposure cohorts. Additionally, STAT3 exhibited a negative causal relationship with the occurrence of PCOS, suggesting that it may act as protective factor for PCOS. Conversely, genetically predicted plasma CCL22 levels and CCL5 were positively associated with PCOS risk, indicating that CCL22 and CCL5 may serve as risk factors for the development of PCOS.

### Causal effects of cytokines on PCOS (replication)

**Fig. 3:**
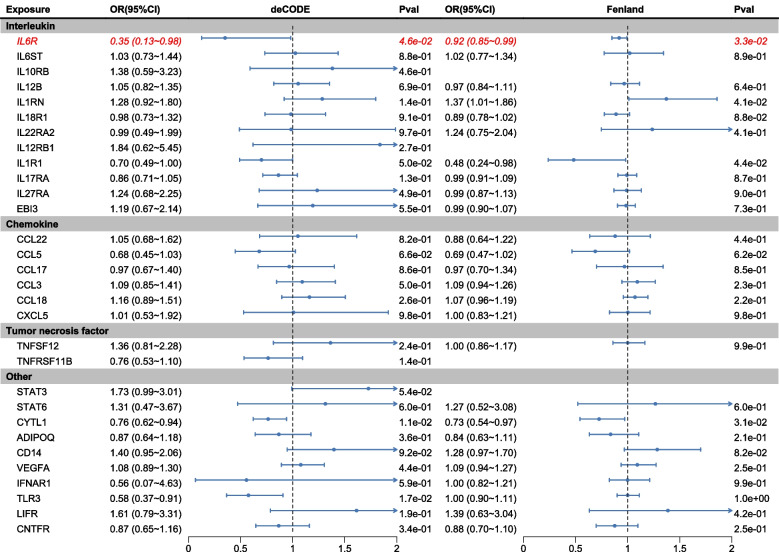
Summary of MR estimates of cytokine (Fenland) on the risk of PCOS. Forest plots illustrating the causal relationships and effect sizes between various types of cytokines from the Fenland database and PCOS. The OR (OR) was calculated using the fixed effect IVW method. The horizontal bars represent the 95% confidence intervals (CI). The squares in the plot represent the OR values, and the horizontal lines represent the confidence intervals. If the confidence intervals extend beyond the range of 0.5–1.5, they are indicated by arrows

Subsequently, we conducted another MR analysis using the FinnGen for PCOS GWAS (1424 cases, 179,322 control) to further minimize bias. When utilizing deCODE as the exposure, the results demonstrated that IL6 still exhibited a significant negative causal relationship with PCOS, with a substantial effect size (IVW: OR = 0.35, 95% CI: 0.13–0.98, *P* = 4.62e − 2) (Fig. 3 and Additional file 1: Table S6). Additionally, CYTL1 and TLR3 were found to have a significant negative causal relationship with PCOS (CYTL1: IVW: OR = 0.76, 95% CI: 0.62–0.94, *P* = 1.13e − 2; TLR3: IVW: OR = 0.58, 95% CI: 0.37–0.91, *P* = 1.73e − 2) (Fig. 3 and Additional file 1: Table S6). To address potential data biases, we conducted a validation analysis utilizing 33 cytokines from the Fenland plasma protein cohort as exposure variables for MR analysis. The results demonstrated that inverse causal association between plasma IL6R levels and PCOS risk (WR: OR = 0.92, 95% CI: 0.85–0.99, *P* = 3.31e − 2) (Fig. 3 and Additional file 1: Table S7). Additionally, the protective effect of CYTL1 plasma levels against PCOS risk was reaffirmed (IVW: OR = 0.73, 95% CI: 0.54–0.97, *P* = 3.15e − 2). Notably, a novel finding emerged, revealing an inverse causal relationship between IL1R1 plasma levels and PCOS risk (WR: OR = 0.48, 95% CI: 0.24–0.98, *P* = 4.44e − 2). Conversely, genetically predicted IL1RN plasma levels exhibited a positive causal association with PCOS onset (IVW: OR = 1.37, 95% CI: 1.01–1.86, *P* = 4.15e − 2). Other cytokines yielded no significant causal effect on PCOS.

In summary, our findings underscore the protective roles of IL6R and CYTL1 plasma levels against PCOS, while highlighting IL1RN as potential risk factors. Specifically, genetically predicted plasma IL6R levels exhibit a negative causal impact on PCOS risk, implicating that IL6R is a protective factor against PCOS development. Conversely, IL1RN are associated with increased PCOS risk, underscoring their potential as risk factors.

### Colocalization analysis

Despite the efficacy of MR in mitigating bias stemming from confounding factors, the presence of linkage disequilibrium (LD) between single nucleotide polymorphisms (SNPs) can lead to spurious associations. This phenomenon arises when SNPs in LD at the same locus influence protein levels and outcomes through distinct pathways. To ascertain whether the genetic association observed between IL6R and PCOS arises from shared SNPs or not, we conducted a colocalization analysis.

**Fig. 4:**
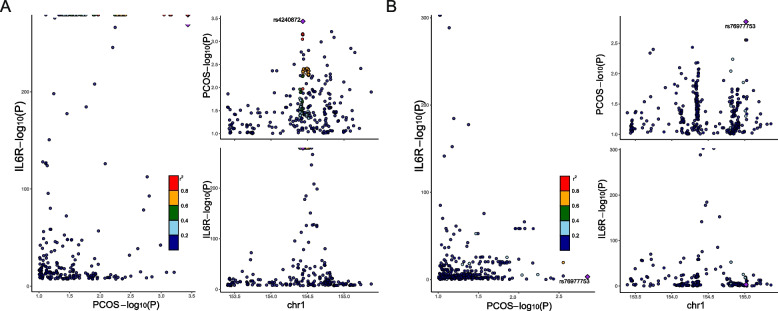
Colocalization analysis between IL6R and PCOS. The scatter plot depicts the gene loci shared by IL6R and PCOS and their chromosomal locations. The diamond represents the locus with the highest correlation. **A** the GWAS data for APCOS is from the Tyrmi JS et al. article; **B** the GWAS data for PCOS is from FinnGen

Our colocalization analysis of IL6R and PCOS yielded robust genetic evidence supporting colocalization, with posterior probabilities (PP4) exceeding 80% under various priors (Fig. 4, Additional file 1: Table S8). These findings suggest the presence of common causal variants influencing both IL6R levels and PCOS susceptibility, with a high degree of certainty.

### Summary-data-based MR (SMR) analysis

In order to validate our findings, we conducted a Summary Data-based Mendelian Randomization (SMR) analysis along with HEIDI tests. IL6R demonstrated significant results in both the SMR test (*P* = 0.035) and the HEIDI test (*P* = 0.237), indicating a robust genetic correlation between IL6R and PCOS. Further details are provided in Table S9.

### Healthy lifestyle guidance

**Fig. 5:**
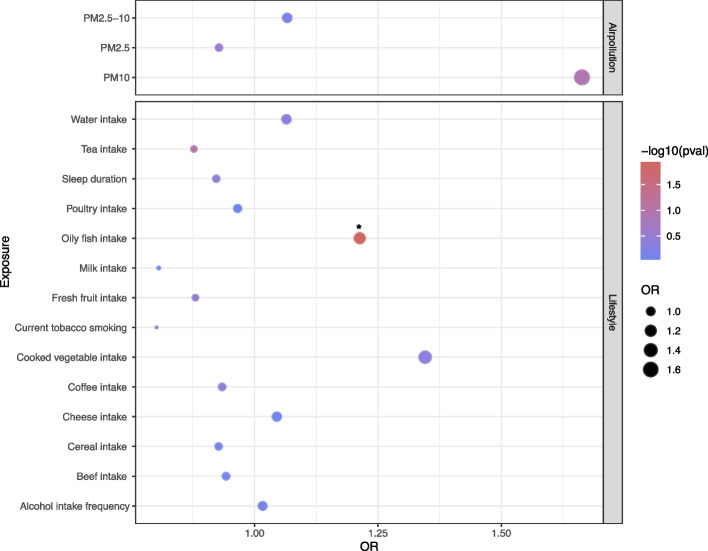
Mendelian randomization for healthy lifestyle factors and IL6R. The bubble shows the causal relationship between 14 lifestyles and 3 air pollutions and IL6R, * < 0.05

In the Mendelian analysis of 14 lifestyle factors and 4 air pollutants, only oily fish intake showed a significant positive causal relationship with IL6R expression (Fig. 5, Additional file 1: Table S12). This suggests that increasing the intake of oily fish in the diet may reduce the risk of PCOS.

## Discussion

This MR study investigated the relationships between 1,125 plasma proteins and the risk of PCOS. Among these proteins, we specifically examined 33 cytokines believed to have a potential causal link with PCOS. Notably, only one plasma protein, IL-6R, was consistently shown to have a significant negative effect on PCOS risk.

Our MR analysis revealed an inverse association between genetically predicted IL6R levels and PCOS risk. Numerous studies have demonstrated that PCOS is a multifactorial disease characterized by systemic inflammation, dysregulation of steroid hormones, and autoimmune components [37]. However, the relationship between increased soluble IL-6R (sIL-6R) levels (which generally imply more IL-6 trans-signaling and thus a stronger pro-inflammatory effect) and a reduced incidence of PCOS remains controversial.

Through extensive literature research, we found that the rs2228145 polymorphism of sIL6R exhibits a protective effect against various human inflammatory diseases [38]. Two initial meta-analyses attributed this protective effect to a reduction in IL-6 classical signaling on hepatocytes, leading to decreased production of the acute phase protein CRP. These studies suggested that homozygous carriers of the rs2228145 minor allele displayed less membrane-bound IL-6R on their cell surface, consistent not only with increased proteolytic cleavage but also with reduced acute phase protein production.

More recent studies proposed an additional explanation, suggesting that the anti-inflammatory buffer system composed of sIL-6R and soluble gp130 (sgp130) functioned differently. In healthy individuals, blood levels of IL-6 range from 1–5 pg/ml but can increase by approximately 1,000-fold in inflammatory states. Steady-state levels of sIL-6R range from 25–75 ng/ml and can increase 2 to threefold during inflammatory diseases, while sgp130 levels remain constant at 250–400 ng/ml. During inflammation, IL-6 secreted by cells binds to sIL-6R with high affinity. The IL-6/sIL-6R complex subsequently binds to sgp130, neutralizing its activity. Thus, sIL-6R and sgp130 in the blood act as a buffer for IL-6, preventing excessive stimulation. However, because sgp130 levels exceed those of sIL-6R, the buffer's capacity is determined by the level of sIL-6R [39].

**Fig. 6:**
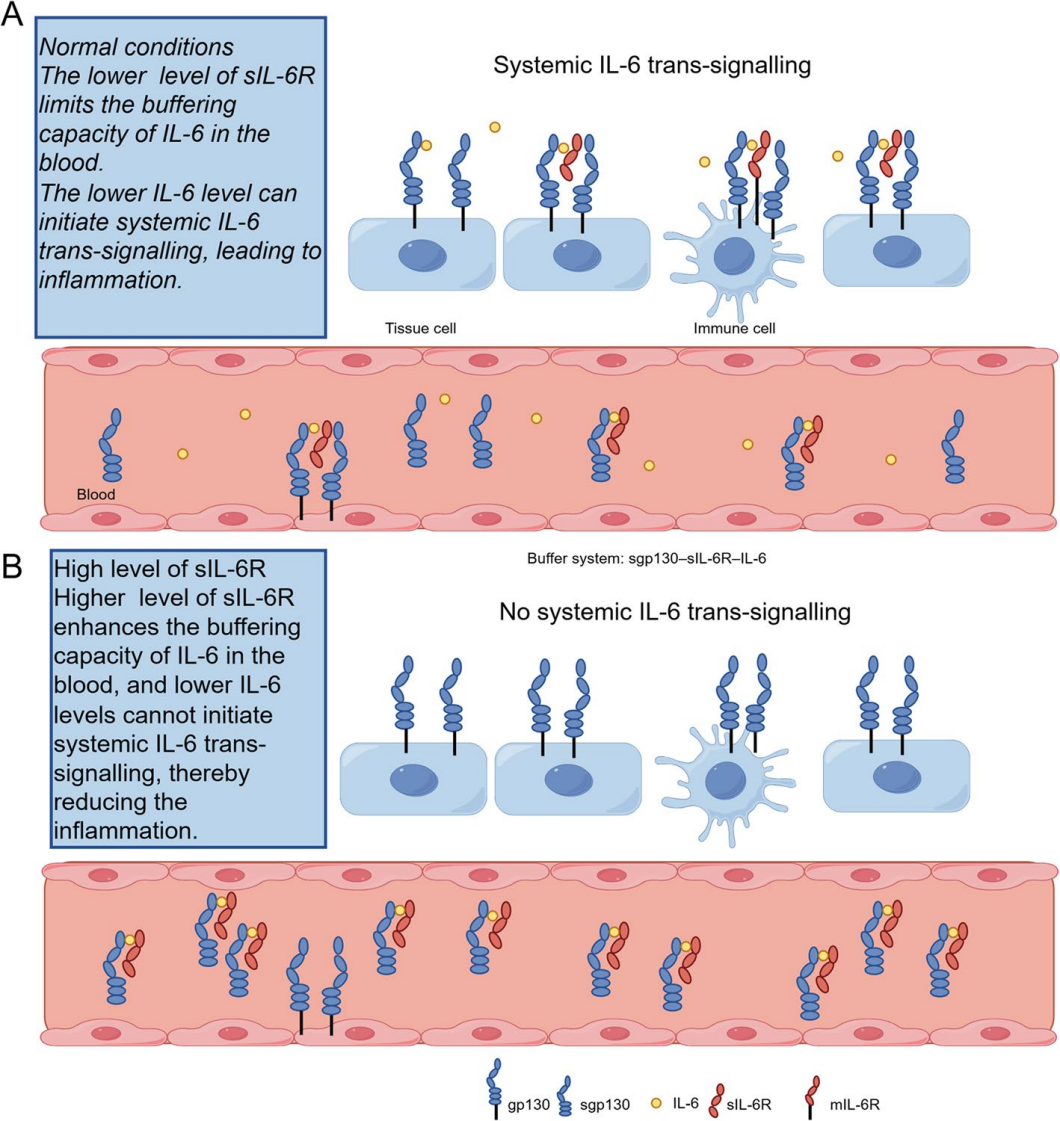
The IL-6 buffer in the blood. The IL-6 bufer in the blood is formed by sIL-6R and sgp130. **A** Normal sIL-6R levels and low IL-6 levels will cause IL-6 trans-signaling pathway; **B** High sIL-6R levels and low concentrations of IL-6 levels were neutralized by the buffer system

This IL-6 buffer system, composed of IL-6/sIL-6R/sgp130, better explains our findings (Fig. 6). Under normal conditions, secreted IL-6 is neutralized by this buffer system, preventing trans-signaling. Once IL-6 levels exceed the buffer capacity, IL-6 disperses through the blood into various tissues, triggering an inflammatory cascade. To validate our conclusion, we further investigated the impact of the sIL6R instrumental variables (IVs) on CRP levels. Our results showed that genetically predicted high sIL6R expression is significantly associated with reduced CRP expression, consistent with the buffer system hypothesis (Additional file 1: Table S9). Genetically predicted high sIL6R expression enhances the blood's IL-6 buffer system, resisting inflammation and thereby reducing the incidence of PCOS.

In addition to finding that IL6R plasma levels are a protective factor for PCOS, we also explored that the level of CCL22 in plasma has a positive causal relationship with PCOS. CCL22 is a macrophage-derived immunosuppressive chemokine, also known as macrophage-derived chemokine (MDC) [40]. Given the inflammatory component of PCOS, altered CCL22 levels could influence the balance between pro-inflammatory and anti-inflammatory immune responses, potentially exacerbating the chronic low-grade inflammation observed in PCOS patients. Additionally, CCL22 is known to be induced by IL-4 and IL-10 signaling, which are involved in Th2 immune responses and metabolic regulation [41]. Dysregulation of these pathways has been implicated in insulin resistance and ovarian dysfunction, which are key features of PCOS [42, 43]. Our results align with emerging evidence linking immune dysregulation to PCOS pathogenesis, suggesting that CCL22 may play a role in shaping the ovarian and systemic inflammatory environment. Although experimental validation is needed, these findings highlight a possible immunometabolism relation between CCL22-mediated immune regulation and PCOS risk. There is no specific inhibitor for CCL22 in clinical practice, but research has been explored for strategies to modulate its activity. For example, fucoidan, a sulfated polysaccharide, has been shown to inhibit CCL22 production in M2 macrophages via the NF-κB pathway, suggesting potential therapeutic applications. Additionally, CCL22-based peptide vaccines have been investigated for their ability to induce anti-cancer immunity by modulating the tumor microenvironment, highlighting the role of this chemokine in immune regulation. While these studies provide insights into CCL22 modulation, further research is necessary to develop specific CCL22 inhibitors and evaluate their therapeutic potential in PCOS [44].

Then our findings indicate that IL1R1 serves as a protective factor for PCOS, whereas IL1RN appears to increase PCOS risk. This contrast reflects the complex regulatory mechanisms of the IL-1 signaling pathway. As a key player in inflammatory and metabolic regulation, IL-1 signaling exhibits complex and even paradoxical effects in PCOS pathophysiology. Studies have shown that PCOS patients exhibit elevated levels of IL-1 receptor antagonist (IL-1Ra), which are strongly correlated with obesity and metabolic dysfunction. Notably, even after adjusting for BMI, higher IL-1Ra levels remain predictive of impaired glucose metabolism, as evidenced by oral glucose tolerance test (OGTT)outcomes [45]. This suggests that IL1RN-mediated inhibition of IL-1 signaling may contribute to metabolic disturbances, potentially exacerbating insulin resistance and systemic inflammation. In contrast, IL1R1, the receptor for IL-1 cytokines, has a more complex role. While it is typically associated with pro-inflammatory responses, its protective association with PCOS risk in our study suggests that some degree of IL-1 signaling might be necessary for immune and metabolic homeostasis [46]. The interplay between IL1R1 and IL1RN highlights the fine-tuned regulation of the IL-1 pathway, where excessive inhibition (via IL1RN) could disrupt metabolic balance, while controlled activation (via IL1R1) might be beneficial. These findings underscore the need for further research to clarify how IL-1 signaling dynamics influence PCOS pathogenesis, particularly in the context of immune-metabolic interactions.

Finally, we hope that we can reduce the risk of PCOS by adjusting our lifestyle or reducing the intake of air pollutants. The analysis shows that the intake of oily fish can significantly increase the expression level of IL6R to reduce PCOS. Through literature survey, we found that the largest nutrient contribution of oily fish is lipids, which play a beneficial role in preventing many diseases, including cardiovascular diseases such as stroke and acute myocardial infarction. They also help prevent neurological, metabolic and immune system-related diseases, as well as support weight control. The consumption of oily fish is also important at different stages of human life from conception to old age [47]. Therefore, we may remind patients to consume more oily fish to reduce the risk of PCOS in clinical life guidance. Future studies should explore whether oily fish supplement or other dietary modifications can directly regulate IL6R activity and reduce PCOS risk. Randomized controlled trials assessing the effects of oily fish intake on cytokine levels and metabolic outcomes in PCOS populations would provide valuable evidence. Additionally, longitudinal cohort studies tracking dietary habits, cytokine profiles, and PCOS development could help clarify the long-term impact of dietary interventions on inflammatory pathways related to PCOS.

There are several strengths in our study. Firstly, we systematically explored the associations between plasma proteins and PCOS risk using a proteome-wide MR design. Additionally, we only considered plasma proteins that were discovered and replicated in two different cohorts to be associated with PCOS, ensuring robustness of the findings. Furthermore, by limiting the study to individuals of European ancestry, we mitigated the potential impact of population stratification bias, which can introduce confounding effects into the analysis.

However, several limitations should be acknowledged. Firstly, the sample size of the PCOS cohort was relatively small which may have introduced some bias into the calculations, and should be considered when interpreting the results. Secondly, we must acknowledge the limitations imposed by the small number of IVs. MR-PRESSO requires at least three independent IVs to effectively detect and correct outliers, which was not met in our dataset. To address the issue of pleiotropy, we instead performed MR-Egger intercept tests and Cochran’s Q tests to assess directional pleiotropy and heterogeneity. These analyses did not indicate substantial pleiotropy, supporting the validity of our causal estimates. In addition, we replicated our findings using an independent GWAS dataset, further strengthening the robustness of our results. These limitations underscore the importance of cautious interpretation and application of our findings.

## Conclusions

Collectively, this study highlights IL6R as a protective factor in PCOS and offers novel insights into the molecular underpinnings and potential therapeutic avenues. Additionally, the identified biomarkers CCL22 may hold promise for future use as early screening biomarkers for polycystic ovary syndrome.

## Supplementary Information

Additional file 1

## Data Availability

No datasets were generated or analysed during the current study.
